# Exploring the relationships between health literacy, social support, self-efficacy and self-management in adults with multiple chronic diseases

**DOI:** 10.1186/s12913-023-09907-5

**Published:** 2023-08-30

**Authors:** Thi Thuy Ha Dinh, Ann Bonner

**Affiliations:** 1 School of Nursing, University of Tasmania, Launceston, TAS Australia; 2 School of Nursing and Midwifery, Griffith University, Brisbane, QLD Australia; 3 Kidney Health Service, Metro North Hospital and Health Service, Brisbane, QLD Australia

**Keywords:** Health literacy, Multimorbidity, Comorbidity, Multiple chronic diseases, Social support, Self-efficacy, Self-management

## Abstract

**Background:**

Self-management in chronic diseases is essential to slowing disease progression and preventing complications. However, empirical research on the associations of critical factors, such as health literacy, social support, and self-efficacy with self-management in the context of multiple chronic diseases is scarce. This study aimed to investigate these associations and provides insights for healthcare providers to develop effective educational strategies for people with multiple chronic diseases.

**Methods:**

Using a cross-sectional survey design, adults (n = 600) diagnosed with at least two chronic diseases were conveniently recruited. To measure health literacy, social support, self-efficacy, and chronic disease self-management behaviours, the Health Literacy Questionnaire (HLQ), Medical Outcome Study - Social Support Survey, Self-efficacy in Managing Chronic Disease, and Self-management in Chronic Diseases instruments were utilized respectively. Comorbidity status was assessed using Age-adjusted Charlson Comorbidity Index (ACCI). A generalised linear regression model was used with a backward technique to identify variables associated with self-management.

**Results:**

Participants’ mean age was 61 years (SD = 15.3), 46% were female, and most had up to 12 years of education (82.3%). Mean scores for HLQ domains 1–5 varied from 2.61 to 3.24 (possible score 1–4); domains 6–9 from 3.29 to 3.65 (possible score 1–5). The mean scores were 52.7 (SD = 10.4, possible score 0–95), 5.46 (SD = 1.9, possible score 0–10) and 82.1 (SD = 12.4, possible score 30–120) for social support, self-efficacy, and self-management, respectively. Mean ACCI was 6.7 (SD = 2.1). Eight factors (age > 65 years, being female, 4 health literacy domains, greater social support, and higher self-efficacy levels) were significantly associated with greater self-management behaviours while comorbidity status was not. The factors that showed the strongest associations with self-management were critical health literacy domains: *appraisal of health information, social support for health*, and *healthcare provider support.*

**Conclusions:**

Developing critical health literacy abilities is a more effective way to enhance self-management behaviours than relying solely on self-confidence or social support, especially for people with multiple chronic diseases. By facilitating communication and patient education, healthcare providers can help patients improve their critical health literacy, which in turn can enhance their self-management behaviours.

## Background

As the global population continues to age, there has been a significant rise in the prevalence of multiple chronic diseases among older individuals, which is becoming a growing burden on healthcare systems worldwide [1]. People with multiple chronic diseases are likely to experience extra physical and psychological symptoms, requiring a greater frequency of visits to a range of healthcare professionals, higher medication burden, and more hospital admissions [2, 3]. Developing self-management behaviours is crucial for them to prevent long-term health complications [4], reduce or slow chronic disease progression [5], and improve health-related quality of life [6]. Except for unique requirements in treatment, adequate self-management in any chronic disease would involve six core skills - problem-solving, decision-making, resource utilisation, establishing and maintaining a patient-provider relationship, action planning, and self-tailoring to manage the diseases [7]. Self-management can be more challenging for individuals with low health literacy capabilities and is often reflected in lower adherence to treatment regimens [8], increasing difficulty with accessing suitable health services, and higher rates of emergency department presentations [9].

Health literacy comprises the ability to obtain, communicate, process and understand health information and services, and to make decisions about one’s health [10]. Health literacy is more than simply the ability to read health information as functional literacy is not sufficient for people to manage health conditions or complex healthcare systems. Nutbeam proposed the notion that health literacy is inclusive of three ascending hierarchical skill domains: (i) *functional* – reading and writing skills, (ii) *communicative* – cognitive and literacy skills needed to apply information and (iii) *critical* – to analyse information and to use that information to make health decisions [11]. Studies of health literacy across demographic groups have demonstrated that many people have difficulties in at least one of those health literacy domains [12], especially those with multiple chronic diseases [13]. Research has shown that different domains of health literacy have varying impacts on self-management behaviours in chronic diseases. While it is important for individuals to be able to understand basic health information, functional health literacy has been found to be less crucial than communicative and critical health literacy domains [14] in fostering self-management behaviours in chronic diseases [15–19].

While strong health literacy abilities empower individuals to make their own health decisions, for various reasons, such as being older or having multiple diseases, can mean that many people are likely to need some sort of support from a network of social relationships (e.g. family members, friends, neighbours, relatives and others) to do so [20]. These social relationships can provide various types of support around everyday activities, and this support is more important when someone needs help with healthcare. Access to social support networks can improve mental health [21] and greater adherence to a treatment plan [22], both of which are core to successful self-management behaviours. Social support can come in variety of forms, inclusive of emotional, informational and tangible support [23]. For example, Wang et al. tested a model finding that informational support, over other social supports, was the most important in type 2 diabetes self-management [24]. Social support also has a strong association with chronic kidney disease (CKD) self-management than functional health literacy does [14].

Another contributing factor to self-management is self-efficacy, which is the self-belief of an individual about their capacity to successfully perform an activity [25]. Self-efficacy is a mechanism to achieve effective self-management, as it is a core belief that underlies each of the basic processes of behavioural changes: it’s the motivation to change, the ability to overcome obstacles, and the extent one can maintain new behaviour [26]. Studies consistently report a positive correlation between self-efficacy and chronic disease self-management [27, 28]. Self-efficacy strategies when integrated into interventions as a mechanism to improving chronic disease self-management behaviours, have demonstrated effectiveness [29, 30].

The literature indicates that health literacy, social support, and self-efficacy individually or collectively have positive associations with chronic disease self-management abilities. There have been studies examining the individual relationships between health literacy [31] or social support [32] and self-management, or both health literacy and social support as predictors of self-management [14, 33]. Some of these studies have been flawed with respect to health literacy through only measuring the functional dimensions of health literacy [14]. Most of these studies examined these relationships in one chronic disease [31–33]. Due to the increasing prevalence of and burden associated with living with multiple chronic diseases, people will need greater health literacy capabilities, self-efficacy, and/or need additional social support. However, no studies have examined the relative strengths of the relationships between all these concepts on self-management behaviours for multiple chronic diseases. This knowledge would inform healthcare providers to better support patients.

### Aims

This study aimed to test the associations between health literacy, social support, and self-efficacy on self-management behaviours of individuals with multiple chronic diseases.

## Methods

### Design

A cross-sectional design was employed and is reported according to STROBE guidelines [34].

### Participants and setting

Participants were recruited from three inpatient medical wards (cardiology, nephrology, and endocrinology) at a large tertiary public hospital in Hanoi, Vietnam between November 2018 to April 2019. The inclusion criteria were: (i) being ≥ 18 years of age, (ii) having a confirmed medical diagnosis of 2 or more of the following chronic diseases: heart failure with a New York Heart Association classification of II-IV [35], hypertension (or systolic BP ≥ 140 mmHg, diastolic BP ≥ 90 mmHg or on prescribed antihypertensive agents) [36], CKD with an estimated glomerular filtration rate (eGFR) < 90mL/min/1.7m^2^ (or eGFR grades 2–5 according to Kidney Disease Improving Global Outcomes guidelines) [37] or diabetes type 2 [38] for at least 3 months, and (iii) volunteered to participate. A medical doctor in each ward identified patients with two or more of these diseases/conditions and notified the research team. These chronic diseases were identified due to their high prevalence and high frequency of their combinations in clinical settings, and their similar principles of self-management, inclusive of monitoring symptoms, managing medication treatment, and making dietary, exercise and other lifestyle adjustments [39], that enabled the measurement of self-management using the same instrument, purposefully modified for this study. Excluded where those with a critical illness, cognitive impairment, or unable to communicate verbally.

### Sample size

Sample size was calculated using a rule-of-thumb ratio of at least 10 participants per variable in a structural equation model with 44 variables [40], and adding a further 20% to account for missing data, resulted in a minimum sample size of 528 needed for this study.

### Data collection

Following ethics approval and obtaining site permissions, a medical doctor in each ward/clinic assisted with identifying eligible patients and referred them to the research team. Four research assistants extracted data from patients’ medical records and assisted participants with responding to a printed set of instruments by explaining or reading questions aloud. Research assistants were final year undergraduate nursing students and were trained in participant recruitment, obtaining consent, and collecting data. Data collection occurred at bed sides or in a quiet place in outpatient clinics. It took participants approximately 20–30 min to complete all instruments.

### Instruments

#### Demographic questionnaire

A brief demographic questionnaire gathered data on age, gender, marital status, highest qualification, employment status, average household income and who was main support person. Data was also extracted from participants’ medical records, including all medical diagnoses for the calculation of Charlson Comorbidity index.

#### Aged-adjusted charlson comorbidity index (ACCI)

The comorbidity status was assessed using the Age-adjusted Charlson Comorbidity Index (ACCI), which was calculated as a sum of a participant’s score for age and for chronic disease/condition [41]. Age is scored 1 point for every decade from the age of 40. Each chronic disease is scored on a scale ranging from 1 to 6 depending on the severity of the condition.

#### Health literacy questionnaire (HLQ)

Health literacy was measured using the HLQ, a multidimensional instrument comprising 44 items covering 9 conceptually distinct domains of health literacy. Each item is scored by a 4-point Likert scale (domains 1–5; 1 = strongly disagree to 4 = strongly agree) or a 5-point Likert (domains 6–9; 1 = cannot do or always difficult to 5 = always easy). A mean score is calculated for each domain, and no aggregated score is computed for overall scale. A cut off score of ≤ 2.5 (domains 1–5) and ≤ 3.5 (domains 6–9) indicates a low health literacy level, as recommended by the instrument developers. Descriptions of a high and low level of health literacy in each domain have been stated elsewhere [42]. The domains also account for Nutbeam’s three health literacy dimensions of functional (domains 2, 8, 9), communicative (domains 1, 3, 4, 6, 7, 8) and critical (domains 3, 4, 5) abilities. Unlike other functional health literacy instruments, the HLQ also measures the skills needed to access and use health services (i.e. navigating in health setting, engaging with healthcare providers, etc.) and how responsive the health system and professionals are to them [43]. The HLQ demonstrates good composite reliability of each scale [42] and confirmatory factor analysis demonstrates each of the nine HLQ domains as a robust independent measure [44]. The HLQ is available in many languages including Vietnamese, and permission to use it was granted from the instrument developers. In this current study, Cronbach’s alpha coefficients of HLQ domains varied from 0.81 to 0.89.

#### Medical outcomes study – social support survey (MOS-SS)

The extent of social support a person receives from their social network was measured using the MOS-SS [23]. This instrument consists of one item asking about the number of supporters that a person has when needed, and 19 other items are rated on a Likert scale from 1 (none of the time) to 5 (all of the time) which measures social support experience in 4 dimensions: emotional/information support, tangible support, affectionate support, and positive social interaction. A total score is calculated by a sum of all individual items; a higher score indicates a greater level of perceived social support. This instrument was originally developed for people with chronic diseases, and it had been translated into the Vietnamese language and used in several studies in Vietnam demonstrating good construct validity and reliability [45, 46] including this current study (Cronbach’s alpha was 0.95).

#### Self-efficacy for managing chronic disease (SEM-CD)

The SEM-CD is used to assess how much confident an individual feels in undertaking self-management tasks in chronic diseases [47]. It has 6 items related to confidence in managing (1) fatigue, (2) physical discomfort/pain, (3) emotional distress, (4) other symptoms/health problems, (5) tasks/activities needed to manage health conditions, and (6) things other than just taking medication. Each item is rated from 1 (not confident at all) to 10 (totally confident); scale scores are standardised into a mean between 1 and 10 with higher scores representing greater confidence in chronic disease self-management. The SEM-CD has been translated into Vietnamese using forward and backward process, and demonstrated a good Cronbach’s alpha of 0.98 in this current study.

#### Chronic disease self-management instrument (CD-SM)

The search of literature revealed none of the existing self-management instruments were suitable for multiple chronic diseases. Despite the uniqueness in causes and treatment, self-management in diabetes, cardiovascular diseases, and CKD do share the six core self-management skills [7], therefore, an existing Vietnamese language instrument measuring CKD self-management behaviours was modified for this project [48]. This instrument consists of 30 items rated by a 4-point Likert scoring scale from 1 = never to 4 = always; a possible score is between 30 and 120. The instrument measures the frequency of behaviours performed in 4 subscales ‘Understanding my chronic disease’, ‘Taking action to manage my chronic disease’, ‘Seeking social support’ and ‘Diet adherence’. Phrases were amended to suit multiple conditions rather than only CKD without alterations in self-management behaviours, for example the word “kidney disease” was replaced by “my diseases”. Confirmatory factor analyses (CFA) using this current study data demonstrated that each subscale of the CD-SM was reliable (Cronbach’s alpha coefficients: 0.79–0.86) and valid (*Understanding my chronic disease*: X2/df = 4.60, CFI = 0.94, RMSEA = 0.78 [90% CI 0.07, 0.08]; *Taking action to manage my chronic disease*: X2/df = 5.46, CFI = 0.96, RMSEA = 0.08 [90% CI 0.07, 0.10]; *Seeking social support*: X2/df = 1.76, CFI = 0.99, RMSEA = 0.04 [90% CI 0.00, 0.07]; *Adhering to a healthy diet*: X2/df = 10.1, CFI = 0.99, RMSEA = 0.12, [90% CI 0.06, 0.19]). For reference, a good-fit CFA model, which reflects instrument structural validity, is when CFI ≥ 0.95 and RMSEA falls into [0.4–0.6] [49].

### Ethical considerations

This study was approved by a human ethics committee (Queensland University of Technology, approval number 1800001035). Participants received both verbal and written explanation of the study prior to providing voluntary consent to participate, and they had the right to skip any item they did not want to answer, and or to withdraw their participation from the study at 2 weeks after returning their completed questionnaires to the research team.

### Statistical analysis

Data were inputted into IBM SPSS™ statistics version 23.0 (IBM Corp., 2015) and assessed for missing data, distributions, and outliers. There was no missing data in the HLQ, MOS-SS, SEM-CD and CD-SM. Scores of HLQ domains, MOS-SS, SEM-CD and CD-SM and ACCI were calculated according to instrument instructions. The cut-off points determining a low health literacy were inconsistent between studies, so we used cut-off scores of ≤ 2.5 (domains 1–5) and ≤ 3.5 (domains 6–9) as suggested by the instrument’s developers. The proportion of people with low health literacy was calculated for each HLQ domain. ACCI scores were divided into 2 groups of < 6 and ≥ 6 (due to an ACCI mean = 6.68). Descriptive statistics were calculated for measured variables (means, standard deviations for the continuous and numbers, percentages for the categorical). Independent t-tests were used to examine the differences between demographic groups in health literacy, self-management, social support, and self-efficacy. Pearson’s correlation coefficients were used to test the linear relationships between these outcome variables. Internal consistency reliability was tested for each measurement scale using a Cronbach’s alpha coefficient.

To determine which factors had a significant association with self-management behaviours, a generalised linear regression model was used. All assumptions for this model were met, including independent and random data, normal distribution of self-management variables through standard residual plots and linear relationships between variables. Five demographic variables were significantly associated with self-management (p < 0.05) and were used in the model - gender (*Male/Female*), age *(< 65*, ≥ *65*), education (*VET/University/Higher degree; Up to year 12*), residential area (*City/urban; Rural/remote/mountainous*), and main support person (*Spouse/Children; Others).* The severity of comorbidity, assessed by ACCI, was not significantly associated with self-management, so was not included in the multivariate analysis. The HLQ domain 3 (*Actively managing my health*) has statements partly overlapping with the concept of self-management, so no correlation was sought between it and the self-management variable, this domain was excluded from multivariate analysis. Five demographic, 8 HLQ domains (except domain 3), self-efficacy and social support variables were entered in the model. Education, residential area, main support person and HLQ domains 6, 7, 8, 9 variables were not significant in the model and were excluded. Standardised coefficients of each variable, 95% confidence intervals (CI), and p values of the final model were reported. The magnitude of standardised coefficients of each factor was compared to determine which factors had higher association with self-management.

## Results

### Participants’ characteristics

A total of 600 participants with a mean age of 61.54 (SD = 15.3 years) ranging from 20 to 89 years were recruited. Most were male (54%), married (84%), had 12 years of school education or lower (82.4%), and about half lived in a rural area (56.4%). Almost two-thirds (62.5%) reported a monthly family income of 5 million VND or lower (~ US$ 215). All participants had several chronic diseases; the most common chronic diseases were hypertension (89%), diabetes (62%), CKD (61%), and heart failure (28%). The mean ACCI was 6.7 (SD = 2.1), and 71.3% had an ACCI score ≥ 6 (see Table 1).

**Table 1:** Participant characteristics (n = 600)

Characteristics	Number	Percentage
**Age group**, ≥ 65 years	293	48.8%
**Gender**, Male	326	54.3%
**Marital status**, married	502	83.7%
**Rural residents**	338	56.3%
**Education**, Year 12 or lower	494	82.3%
**Monthly income**, ≤ 5 million VND ($US 215)	395	65.8%
**Main support person** (spouse or children)	488	81.3%
**Chronic disease diagnosis^**		
Heart failure	170	28.3%
Hypertension	537	89.5%
Diabetes	375	62.5%
Chronic kidney disease	367	61.2%
**Comorbidity index (ACCI)**		
Mild-moderate (0–5)	172	28.7%
Severe (≥ 6)	428	71.3%
**Age**, mean, SD	61.5 (15.3)	
**ACCI**, mean, SD	6.7 (2.1)	

**^** total proportion over 100% due to comorbidity. ACCI: Age-adjusted Charlson Comorbidity Index. VND: Vietnam Dong. $US: United State dollar

### Self-management, social support, self-efficacy and health literacy profiles

The mean scores were 82.1 (CD-SM, SD = 12.4, possible score 30–120), 52.7 (MOS-SS, SD = 10.4, possible score 0–95), and 5.46 (SEM-CD, SD = 1.9, possible score 1–10) indicating that participants had a moderate level of self-management while perceiving a low to moderate level of social support and self-efficacy (see Table 2).

**Table 2:** Descriptions of self-management, self-efficacy, social support and HLQ scores (n = 600)

Variables	Number of items	Possible score	Mean, standard deviation	95% CI	Cronbach alpha	Proportion of low health literacy
Self-management	30	30–120	82.1 (12.4)	81.0, 83.0	0.92	N/A
Understanding my disease	11	11–55	27.9 (5.2)	27.4, 28.3	0.86	N/A
Taking actions to manage my disease	11	11–55	32.20 (5.04)	31.8, 32.6	0.81	N/A
Seeking social support	5	5–25	12.97 (2.68)	12.8, 13.2	0.79	N/A
Adhering to a healthy diet	3	3–15	9.01 (1.87)	8.9, 9.2	0.86	N/A
Self-efficacy	6	1–10	5.46 (1.9)	5.3, 5.6	0.98	N/A
Social support	19	0–95	52.7 (10.4)	51.9, 53.6	0.95	N/A
HLQ domains						
HLQ1. Feeling understood and supported by healthcare providers	4	1–4	2.61 (0.51)	2.57, 2.65	0.83	312 (52%)
HLQ2. Having sufficient information to manage my health	4	1–4	2.71 (0.50)	2.67, 2.75	0.84	216 (36%)
HLQ3. Actively managing my health	5	1–4	2.94 (0.44)	2.91, 2.98	0.83	83 (13.8%)
HLQ4. Social support for health	5	1–4	3.24 (0.42)	3.21, 3.28	0.81	22 (3.7%)
HLQ5. Appraisal of health information	5	1–4	2.62 (0.48)	2.58, 2.66	0.84	240 (40%)
HLQ6. Ability to actively engage with healthcare providers	5	1–5	3.65 (0.59)	3.61, 3.70	0.89	198 (33%)
HLQ7. Navigating the healthcare system	5	1–5	3.61 (0.59)	3.56, 3.65	0.89	230 (38.3%)
HLQ8. Ability to find good health information	5	1–5	3.29 (0.76)	3.23, 3.35	0.89	336 (56%)
HLQ9. Understand health information well enough to know what to do	5	1–5	3.29 (0.71)	3.24, 3.35	0.85	336 (56%)

Notes: HLQ: Health Literacy Questionnaire; CI: Confidence Interval; N/A: Non-applicable

The lowest mean scores of health literacy were in domains 1, 2, 5 (2.61, 2.71, 2.62 respectively) and in domains 8 and 9 (both 3.29), indicating that participants had some difficulties with functional, communicative, and critical health literacy skills. Higher proportions of low health literacy were found in domains 1, 8 and 9 (52%, 56%, 56%, respectively; Table 2). Further details of health literacy profiles, including scores and demographic characteristics with higher health literacy have been published elsewhere [13].

### Associations between social support, self-efficacy, and health literacy domains and self-management

All 9 health literacy domains, social support, and self-efficacy were significantly associated with self-management at the bivariate level, and with each other (p < 0.05). The correlation coefficients with self-management were 0.31 (SEM-CD), 0.42 (MOS-SS), between 0.2 and 0.4 (HLQ domains 6,7,8, 9) and 0.4–0.6 (HLQ domains 1, 2, 4, 5; Table 3).

**Table 3:** Pearson’s correlations between variables (n = 600)

	Self-efficacy	Social support	Self-management	HLQ1	HLQ2	HLQ3	HLQ4	HLQ5	HLQ6	HLQ7	HLQ8	HLQ9
Self-efficacy	1											
Social support (general)	0.14**	1										
Self-management	0.31***	0.42***	1									
HLQ1	0.25***	0.32**	0.48 ***	1								
HLQ2	0.24 ***	0.39**	0.54***	0.44**	1							
HLQ3	0.19 ***	0.32**	N/A	0.40**	0.54**	1						
HLQ4	0.35 ***	0.42**	0.51 ***	0.38**	0.46**	0.46**	1					
HLQ5	0.30***	0.31**	0.58***	0.52**	0.66**	0.60**	0.34**	1				
HLQ6	0.16 ***	0.31**	0.37 ***	0.36**	0.45**	0.45**	0.31**	0.52**	1			
HLQ7	0.18***	0.37**	0.35 ***	0.37**	0.44*	0.42**	0.27**	0.52**	0.87**	1		
HLQ8	0.33***	0.34**	0.42 ***	0.34**	0.50**	0.39**	0.25**	0.61**	0.74**	0.77**	1	
HLQ9	0.30***	0.25**	0.29 ***	0.25**	0.39**	0.34**	0.14**	0.49**	0.69**	0.72**	0.86**	1

Notes: HLQ: Health Literacy Questionnaire; *p < 0.05, ** p < 0.01, *** p < 0.001

### Models explaining self-management behaviours

The model explaining self-management comprised 8 variables (gender, age, health literacy domains 1, 2, 4, 5, self-efficacy, and social support). Overall, the model demonstrated higher self-management scores amongst females (3.99 score higher, 95% CI [2.58, 5.39], p < 0.001), and by those aged 65 and over (2.27 score higher, 95% CI [0.87, 3.68], p = 0.002). The health literacy domain “Social support for health” had a stronger association with self-management (coefficient = 5.96; 95% CI [3.92, 8.01]) than the general social support variable (coefficient = 0.18, 95% CI [0.11, 0.26], respectively). Health literacy (domains 1, 2, 4 & 5) had stronger explanatory power in self-management behaviours (coefficient = 2.34–8.41) than the level of self-efficacy (coefficient = 0.58, 95% CI [0.18, 3.68]) or general social support (coefficient = 0.18, 95% [0.11, 0.26]; see Table 4).

**Table 4:** Generalised linear model explaining self-management behaviours (n = 600)

	Standardized coefficient (95% CI)	p-value
Intercept	17.8 (11.8, 23.7)	< 0.001
Gender		
Female vs. Male	3.99 (2.58, 5.39)	< 0.001
Age		
≥ 65 vs. < 65 years	2.27 (0.87, 3.68)	0.002
Self-efficacy	0.58 (0.18, 0.98)	0.004
General social support	0.18 (0.11, 0.26)	< 0.001
Healthcare provider support (HLQ1)	2.65 (0.98, 4.32)	0.002
Having sufficient information (HLQ2)	2.34 (0.38, 4.31)	0.02
Social support for health (HLQ4)	5.96 (3.92, 8.01)	< 0.001
Appraisal of health information (HLQ5)	8.41 (6.35, 10.47)	< 0.001

Notes: HLQ: Health Literacy Questionnaire; CI: Confidence Interval

## Discussion

This study evaluated the degree to which individuals with multiple chronic diseases have engaged in self-management behaviours, and examined several factors that may impact these behaviours, including health literacy, social support, and self-efficacy. Results confirmed that individuals’ self-management behaviours were significantly correlated to the support they perceived from their social network, how confident they were in self-management tasks, and their abilities to seek, obtain and use health information. Overall, four health literacy domains (healthcare providers support, social support for health, having information, and abilities to appraise health information) had the strongest association with self-management behaviours beyond those of general social support or self-efficacy.

Almost one in every two participants in this study experienced difficulties in feeling understood and supported by healthcare providers (domain 1), finding health information (domain 8) and understanding health information (domain 9), and that one in three had difficulties in engaging with healthcare providers (domain 6) and navigating the health system (domain 7). Compared to other studies involving people with chronic diseases [50, 51], greater proportions of low health literacy were found in this study. The health literacy difficulties perceived by participants could be explained by individual, socioeconomic, and system contexts. Most participants lived in rural locations, had limited education and low incomes. In Vietnam, the healthcare system has many challenges such as the shortage of community-based (primary care) medical doctors, the overload of patients, the tendence for people to value tertiary hospitals in large cities, and the financial cost of healthcare for most of the population [52]. These factors lead to a reduced frequency of healthcare seeking and insufficient health information provided by hospital staff. Improving therapeutic communication is a crucial opportunity to help healthcare providers identify and mitigate the limited understanding about chronic diseases by patients. To date, addressing health literacy has not been considered at ministerial or organisational levels in Vietnam, although it is a priority and responsibility for clinicians as well as policy-makers in many developed countries [53].

For people with multiple chronic diseases, understanding each individual disease and the pathological link between diseases, lifestyle changes, and collective treatment regimens to undertake effective self-management is a complex and challenging requirement. We found that four health literacy domains (having sufficient information, a trusting relationship with a regular healthcare provider/s, specific support for healthcare, and being able to resolve conflicting information), support from a social network, and being confident were significantly associated with self-management in multiple chronic diseases. Critical health literacy (domains 4 and 5) in this study, was much more influential on self-management than communicative (domain 1) and functional (domain 2) health literacy dimensions. Overall, we found that health literacy domains have a stronger association with self-management than either social support or self-efficacy. These results were different from the few existing studies which have previously examined the relationship between health literacy and self-management. For instance, Jacobson et al. found no significant relationship between health literacy and self-management in patients with heart failure [54]. On the other hand, Chen et al. [14] reported that social support did exert a stronger influence on self-management than health literacy among those with CKD. However, it is important to approach these conclusions with caution, as these studies only measured functional health literacy and may not have accounted for other dimensions of health literacy that could have a role in self-management. The current study using the contemporary assessment of health literacy, which is multidimensional, showed the importance of having critical thinking abilities to take active healthcare decisions to self-manage health. For example, these abilities enable patients to recognise breathlessness and weight gain, which may indicate increased fluid retention. Subsequently, patients can make informed decisions such as reducing fluid intake, following guidance to take additional diuretics, or seeking medical assistance when necessary.

Mitigating low health literacy necessitates a comprehensive system-wide approach [55], encompassing various organisational and clinical practice changes. Organisational health literacy is where an organisation such as a hospital or community-based facility demonstrates health literacy policies and practices which makes it easier for individuals to navigate the health system and also directs healthcare providers through policies and training to support patients with low health literacy capabilities [56]. Initiatives such as ensuring clarity of information on hospital websites, simplifying health information pamphlets, and providing training to staff on effective health literacy strategies. It is important to recognise that healthcare providers have a crucial bridging role to mitigate for low health literate patients and the complexity of chronic disease treatment regimens by communicating easy-to-understand information. One simple, low-cost health literacy initiative is the teach-back method where information providers ask patients to repeat the information given to them, or to demonstrate back a procedure, and this initiative should be used for all patients regardless of health literacy abilities [57]. In addition, the results in this study suggested that it’s not enough to simply provide patients with health information; they must also be guided on where to locate information specific to their conditions, and which sources are trustworthy amid the abundance of information available online and elsewhere. Many changes can start from changing therapeutic communication, which is fundamentally the responsibility of the healthcare professional, to enable patients to develop abilities to find and analyse health information. With an enhanced level of health literacy, individuals will have the knowledge and skills to determine when and how to act, enabling them to make informed decisions about their health and well-being. Whenever possible, it is crucial to involve family members (or a friend) in conversations to ensure that patients have the necessary support during a decision-making process. By including these supporters, patients could benefit from their perspectives, as well as emotional and tangible support, leading to informed and active participation in decisions regarding their healthcare.

Overall, these results emphasise the importance of health literacy and social support to self-management in multiple chronic diseases, and in particular, the contribution that critical health literacy capabilities make to be able to enact self-management behaviours. Communication, information giving and educational strategies ought to prioritise tailoring of content and delivery methods to match an individual’s health literacy capability. In addition to providing health information, healthcare providers need to guide patients in identifying reliable sources of information related to their specific conditions. Furthermore, involving family members (or friends) in appointments, conversations, and decision-making processes is likely to enhance the patient feeling supported and could also facilitate the development and sustainability of self-management behaviours. Future research has the potential to develop and evaluate policy and/or practice changes aimed at enhancing health literacy at multiple levels, including ministerial (i.e., department of health), organisational (e.g., hospitals, other health services) or community levels (e.g., local councils, lifestyle groups, etc.). This research would facilitate increased awareness among all stakeholders regarding the prevalence of low health literacy capabilities of patients, and empower stakeholders to fulfill their respective roles in mitigating its impacts on disadvantaged individuals and communities.

### Limitations

There are limitations due to the cross-sectional design of this study that all relationships found between variables and self-management should be interpreted as associations rather than as causal relationships. Participants were sought from only one site limiting generalizability; however, this hospital receives patients from the entire north of Vietnam. Lastly, due to the nature of self-reported measures, it is possible that participants either overstated or underestimated their abilities.

## Conclusion

This study found eight significant variables associated with self-management in people with multiple chronic diseases, of these variables, the critical health literacy domains were the most important. Healthcare providers do have a vital role in establishing a positive supportive relationship with patients through improving all forms of communication with patients and treating every interaction as a valuable teaching opportunity. Future educational strategies need to tailor content, delivery methods and resources to suit each individual’s health literacy capability. This education also needs to instruct patients how to identify reliable sources of information and to also involve family members (or friends), so that patients have the necessary supports to self-manage their diseases. Lastly, for Vietnam, there is a need for policies and action plans around health literacy and chronic disease self-management at each of the health system levels (i.e., ministerial, organisational, community) due to the growing chronic disease burden.

## Data Availability

The dataset generated and analysed during this current study is not publicly available due to sharing data was not part of this study’s ethics approval. The corresponding author should be contacted if there is any request relating to this study data.

## List of Abbreviations

CKD: Chronic kidney disease
STROBE: Strengthening the Reporting of observational studies in Epidemiology
ACCI: Age-adjusted Charlson Comorbidity Index
HLQ: Health Literacy Questionnaire
MOS-SS: Medical Outcome Study – Social Support Survey
SEM-CD: Self-efficacy in managing chronic disease
CD-SM: Chronic disease self-management scale
X2/df: Chi-square divided by degree of freedom
CFI: Comparative Fit index
RMSEA: Root mean square error of approximation
CI: Confidence interval
VND: Vietnam dong
SD: Standard deviation
